# Harm Avoidance and Self-Directedness Characterize Fibromyalgic Patients and the Symptom Severity

**DOI:** 10.3389/fpsyg.2016.00579

**Published:** 2016-04-25

**Authors:** Paolo Leombruni, Francesca Zizzi, Marco Miniotti, Fabrizio Colonna, Lorys Castelli, Enrico Fusaro, Riccardo Torta

**Affiliations:** ^1^Department of Neuroscience “Rita Levi Montalcini”, University of TurinTurin, Italy; ^2^Department of Psychology, University of TurinTurin, Italy; ^3^Rheumatology Unit, Azienda Ospedaliero-Universitaria Cittá della Salute e della Scienza di TorinoTurin, Italy

**Keywords:** fibromyalgia, personality, temperament, character, Italian sample

## Abstract

**Objective:** Evidence in the literature suggests peculiar personality traits for fibromyalgic (FM) patients, and it has been suggested that personality characteristics may be involved in patients’ different symptomatic events and responses to treatment. The aim of the study is to investigate the personality characteristics of Italian FM patients and to explore the possibility of clustering them considering both personality traits and clinical characteristics.

**Design:** The study used a cross-sectional methodology and involved a control group. A self-assessment procedure was used for data gathering. The study included 87 female FM patients and 83 healthy females. Patients were approached and interviewed in person during a psychiatric consultation. Healthy people were recruited from general practices with previous telephone contact.

**Main Outcome Measures:** Participants responded to the Hospital Anxiety and Depression Scale, the Temperament and Character Inventory, the Fibromyalgia Impact Questionnaire and the Short-Form-36 Health Survey.

**Results:** FM patients scored significantly different from healthy participants on the Harm avoidance (HA), Novelty seeking (NS) and Self-directedness (SD). Two clusters were identified: patients in Cluster1 (*n* = 37) had higher scores on HA and lower scores on RD, SD, and Cooperativeness and reported more serious fibromyalgia and more severe anxious–depressive symptomatology than did patients in Cluster2 (*n* = 46).

**Conclusion:** This study confirms the presence of certain personality traits in the FM population. In particular, high levels of HA and low levels of SD characterize a subgroup of FM patients with more severe anxious–depressive symptomatology. According to these findings, personality assessment could be useful in the diagnostic process to tailor therapeutic interventions to the personality characteristics.

## Introduction

Fibromyalgia (FM) is a chronic syndrome characterized by widespread musculoskeletal pain, reflecting a sensitization of the central nervous system (Mease, 2005; Mease et al., 2009; Giacomelli et al., 2011; Ablin and Buskila, 2013). Pain, characterized by hyperalgesia and allodynia, is often associated with fatigue, non-restorative sleep, mood and anxiety disorders, and cognitive impairment (Cazzola et al., 2008). Other common comorbidities are syndromes that may share certain pathophysiological mechanisms, including irritable bowel syndrome, tension-type headache/migraine, interstitial cystitis or painful bladder syndrome. Psychiatric comorbidities and psychological distress had also been widely explored: the prevalence of mood and anxiety disorders is significantly higher in FM patients than in the general population. FM occurs significantly more frequently in females, and its prevalence, ranging between 1 and 8%, increases with age (Wolfe et al., 2013).

From a psychosomatic perspective, FM can be considered the result of the intersection of biological, psychological, environmental, and social factors. This diagnostic and therapeutic approach allows clinicians to appreciate the uniqueness of the individual patient in terms of both clinical characteristics and response to treatment (Sarzi-Puttini and Cazzola, 2009). Psychological factors, such as personality traits, may modulate the physiological response to stressors and, as in the case of FM, influence the cognitive processing of musculoskeletal pain (Clauw, 1995; Malin and Littlejohn, 2012). Evidence in the literature suggests peculiar personality characteristics for FM patients (Wolfe et al., 1984; Alfici et al., 1989; Epstein et al., 1999; Hassett et al., 2000; Bradley, 2005), and several studies have shown a higher prevalence of personality disorders among FM patients than among the general population (Rose et al., 2009; Glazer et al., 2010; Uguz et al., 2010). However, these studies have used different instruments with distinct theoretical personality frameworks, and their findings are quite heterogeneous and difficult to compare (Malin and Littlejohn, 2012).

The theory of personality proposed by Cloninger (the psychobiological model of personality) and the questionnaire derived from this theory, the Temperament and Character Inventory (TCI), seem particularly appropriate to investigate the personality characteristics of FM patients given their biopsychosocial perspective (Cloninger, 1987; Cloninger et al., 1993). In fact, the psychobiological model of personality has been suggested as a person-centered paradigm useful to understand patient’s unity of body, mind, and soul. Such a perspective could be particularly appropriate to capture all the aspects involved in the onset and maintenance of psychosomatics diseases (e.g., FM), as its applicability in detecting personological predictors of well-being and ill-being (Fahlgren et al., 2015). The psychobiological model of personality proposed by Cloninger (1987) considers personality as an interaction between temperament (conceived as the more heritable personality component) and character (which is the more learned component). The temperament dimensions are genetically independent and express the stable throughout life adaptive emotional responses and behavioral reactions to life experiences. The character traits express the self-concept and the self-identity as an integral part of human society and of universe.

Previous studies have examined FM patients’ personality traits using the TCI and identified a profile characterized by high Harm avoidance (HA; a temperament dimension; Anderberg et al., 1999) and low Self-directedness (SD; a character dimension; Lundberg et al., 2009). These preliminary results have been confirmed by recent studies (Santos Dde et al., 2011; Gencay-Can and Can, 2012; Garcia-Fontanals et al., 2014). It is not possible to determine whether the traits of personality associated with FM are the cause or rather the effect of the syndrome: the high levels of HA could be interpreted as a predisposing factor, but also as the consequence of a painful condition that results in chronic avoidance behavior. Furthermore, FM patients have been observed to be a non-homogeneous group from a clinical standpoint, and personality dimensions have been hypothesized to be involved in differences in symptomatic events and responses to the treatments (Giesecke et al., 2003; De Souza et al., 2009; Lundberg et al., 2009; Torres et al., 2013). Recently, Torres et al. (2013) suggested that the assessment of FM patients’ personality profile could be useful in subgrouping them to identify vulnerable individuals and to ensure tailored interventions.

In summary, personality characteristics are probably determining factors in genesis, maintenance, and modulation of FM, but only few studies tried to clustering patients on the basis of temperament and character dimensions. The present study tries to partially bridge this gap investigating the possibility of clustering Italian FM outpatients on the basis of their personality characteristics. In particular, study design was built to describe the temperament and character dimensions of these patients and to cluster them considering both personality traits and clinical characteristics.

## Materials and Methods

### Participants and Procedures

The patient participants were females diagnosed with FM who were consecutively recruited at the Clinical Psychology and Psycho-Oncology Unit of the Azienda Ospedaliera e Universitaria “Città della Salute e della Scienza” in Turin, Italy. The diagnoses of FM were made by a rheumatologist according to the ACR 2010 criteria (Wolfe et al., 2010). FM patients previously diagnosed with mood or anxiety disorders, pain due to trauma, general or localized rheumatic diseases, rheumatoid arthritis, inflammatory arthritis, autoimmune diseases or those who were undergoing ongoing treatment with antidepressants were excluded. Participants enrolled in the control group (CG) were recruited from general practices with previous telephone contact. Exclusion criteria for CG participants were a positive psychiatric history, ongoing psychopharmacological treatment or psychotherapy, a positive history of muscle-tension headache, migraine, back pain, or neuralgia, ongoing analgesic treatments and a score ≥16 on the Hospital Anxiety and Depression Scale. The enrollment period for FM patients began in September 2013 and ended in September 2014. During the study period, 87 female patients were observed. None of them met any exclusion criteria. After giving informed consent, all participants were included in the study. The enrollment period for CG participants began in January 2014 and ended in September 2014. During the study period, 99 women were observed and considered for entry in the study. Of these, 5 refused to participate and 11 were excluded according to the aforementioned exclusion criteria. Thus, 83 female subjects gave informed consent and were included in the CG. The study was approved by the appropriate Ethical Review Committee and conducted in accordance with the most recent principles of the Declaration of Helsinki. Before signing their consent, the FM and CG participants were properly informed about the purposes and the methods of the study.

### Measures

The Italian version of the Hospital Anxiety and Depression Scale (HADS) was used to evaluate anxious and depressive symptoms. The HADS assesses anxiety and depression in a not closely psychiatric population (Whelan-Goodinson et al., 2009). The instrument is self-reported and consists of 14 items and two subscales that independently measure anxious symptoms and depressive symptoms. For each subscale, scores of 8–10 are considered borderline and scores above 11 indicate clinical anxiety or depression (Bjelland et al., 2002).

For the personality assessment of the subjects, the Italian version of the TCI was used (Fossati et al., 2001). The TCI is a true-false questionnaire with 240 questions that assess personality by describing aspects of temperament and character. Temperament is considered the more heritable personality component that is stable throughout life and is responsible for adaptive emotional responses and behavioral reactions to life experiences. It is assessed through four dimensions: Harm avoidance (HA), Novelty seeking (NS), Reward dependence (RD), and Persistence (P). In contrast, character is considered the more learned personality component and is thought to mature throughout adulthood and to contribute to the development of one’s self-concept. Character involves differences in values, life choices and aims, and it is assessed through three dimensions: Self-directedness (SD), Cooperativeness (C), and Self-transcendence (ST). Each dimension of temperament and character is further described by several lower-order traits (Cloninger et al., 1994).

Cloninger (1999) released the Temperament and Character Inventory-Revised (TCI-R). The TCI-R contains the same number of items of the original TCI, but differs from it in some aspects: a Likert-type scale (from 1 – definitely false, to 5 – definitely true) has been introduced, instead of the true–false item scale; 51 items have been reformulated, including 5 validity items; furthermore, 4 facets have been introduced both for RD and P dimensions, instead of 1 for P and 3 for RD. In the present study, the original TCI was used because the evidences in the literature we aimed to verify (Santos Dde et al., 2011; Gencay-Can and Can, 2012) used that version.

The Fibromyalgia Impact Questionnaire (FIQ) was used to evaluate the severity of the FM (Burckhardt et al., 1991). The FIQ is a self-report questionnaire that measures the impact of FM on functional capacity and quality of life. It consists of 10 items, and the score ranges from 0 to 100 (where a higher score indicates more severe FM).

The Italian version of the Short-Form-36 Health Survey (SF-36) was used to assess the level of quality of life. The SF-36 consists of 36 questions conceptually related to eight health domains. In the present study, the two main subscales (Physical Component and Mental Component) were considered. Higher scores indicated better quality of life (Apolone and Mosconi, 1998).

### Statistical Analyses

Statistical analyses were performed using SPSS for Mac, version 21.0. Descriptive statistics were calculated to summarize the data. Between-group and between-cluster differences were investigated by performing independent-sample *t*-tests and χ^2^ statistics when appropriate. *K*-means clustering was used for cluster analysis, assuming two clusters a priori. All the TCI dimensions were included in the model. Due to the small sample size, discriminant function analysis was selected to check the goodness of fit of the model that the cluster analysis found and to profile the clusters. All statistical tests were two-tailed with alpha set at 0.05.

## Results

### Sample Demographics

All 87 patients recruited for the study were females who had been diagnosed with fibromyalgia. The mean age of the patients was 52.04 years (*SD* = 10.81). On average, the patients had 18.00 years of schooling (*SD* = 10.62). Regarding marital status, 63 participants (75.9%) were married, 12 (14.5%) were divorced, 6 (7.2%) were single, and 2 (2.4%) were widows. Regarding working status, 45 (54.2%) were employed, 1 (1.2%) was a student, 17 (20.5%) were housewives, 4 (4.8%) were unemployed, 14 (16.9%) were retired, and 2 had missing data. The CG consisted of 83 female participants. The mean age of the participants was 44.31 years (*SD* = 9.16). On average, the patients had 18.00 years of schooling (*SD* = 13.75). Regarding marital status, 65 (78.3%) were married, 2 (2.4%) were divorced, 14 (7.2%) were single and 2 (2.4%) were widows. Regarding working status, 69 (83.1%) were employed, 5 (6.0%) were housewives, 4 (4.8%) were unemployed, and 5 (6.0%) were retired.

### TCI Scores among FM Patients

FM patients scored significantly differently from CG participants on the HA and NS temperament dimensions, and on the SD character dimension. These differences, with the addiction of ST, were also found when comparing FM patients’ TCI scores with normative data provided by Cloninger et al. (1994). Descriptive statistics and significance levels are reported in **Table 1**.

**Table 1:** FM patients’ personality characteristics: comparisons with control group (CG) and normative data.

Variable	FM patients (*N* = 83)	CG (*N* = 83)	Normative data (*N* = 300)
TCI Harm Avoidance (HA)	21.87 ± 6.39	14.87 ± 5.78^b^	12.6 ± 6.8^b^
TCI Novelty Seeking (NS)	16.94 ± 5.29	18.99 ± 5.48^a^	19.3 ± 6.0^a^
TCI Reward Dependence (RD)	15.19 ± 3.38	15.52 ± 3.93	15.5 ± 4.4
TCI Persistence (P)	5.19 ± 1.50	4.95 ± 1.82	5.6 ± 1.9
TCI Self-Directedness (SD)	28.25 ± 6.36	33.52 ± 6.14^b^	30.7 ± 7.5^a^
TCI Cooperativeness (C)	32.93 ± 5.04	33.13 ± 5.90	32.3 ± 7.2
TCI Self-Transcendence (ST)	15.79 ± 5.52	15.51 ± 6.59	19.2 ± 6.3^b^


*FM, Fibromyalgia and TCI, Temperament and Character Inventory. ^a^*p*-value < 0.05. ^b^*p*-value < 0.001.*

### Cluster Characteristics and Between-Cluster Comparisons

On the basis of the TCI dimensions, two clusters were identified by *K*-means cluster analysis. Regarding personality traits, patients in Cluster1 (*n* = 37) were characterized by higher scores on HA and lower scores on RD, SD, and C compared to patients in Cluster2 (*n* = 46). With regard to clinical characteristics, patients in Cluster1 reported more serious fibromyalgia and more severe anxious–depressive symptomatology than patients in Cluster2. Health-related quality of life was better among patients in Cluster2 than among patients in Cluster1. The significance levels of descriptive data and between-cluster differences are fully reported in **Table 2**. Details about demographics are also presented in **Table 2**.

**Table 2:** Demographics, personality characteristics, fibromyalgia severity, anxiety, depression and health-related quality of life: cluster comparisons.

Variable	FM patients Cluster1 (*N* = 37)	FM patients Cluster2 (*N* = 46)	Test of significance

**Continuous**	***m* ± *SD***	***m* ± *SD***	***t*-test**
Age	50.46 ± 9.75	53.30 ± 11.54	-1.195
Education (years)	10.31 ± 3.16	10.87 ± 3.60	-0.717
TCI HA	26.86 ± 4.26	17.85 ± 4.77	8.971^c^
TCI NS	15.81 ± 5.47	17.85 ± 5.02	-1.766
TCI RD	14.22 ± 3.50	15.98 ± 3.10	-2.430^a^
TCI P	5.40 ± 1.36	5.02 ± 1.60	1.159
TCI SD	23.16 ± 4.91	32.35 ± 4.01	-9.384^c^
TCI C	30.43 ± 5.43	34.93 ± 3.68	-4.490^c^
TCI ST	15.92 ± 5.61	15.70 ± 5.51	0.182
FIQ Total Score	67.66 ± 14.80	56.57 ± 17.24	3.100^b^
HADS Total Score	22.05 ± 5.90	13.76 ± 7.45	5.516^c^
HADS Anxiety	11.16 ± 3.83	6.91 ± 3.79	5.048^c^
HADS Depression	10.86 ± 3.14	6.85 ± 4.28	4.761^c^
SF-36 Physical Activity	45.95 ± 20.10	53.04 ± 22.05	-1.516
SF-36 Role-physical	14.53 ± 24.56	22.83 ± 30.20	-1.350
SF-36 Bodily Pain	27.48 ± 18.37	34.06 ± 16.41	-1.723
SF-36 General Health	28.95 ± 19.71	41.78 ± 19.88	-2.935^b^
SF-36 Vitality	26.08 ± 14.87	35.98 ± 16.69	-2.818^a^
SF-36 Social Functioning	40.88 ± 19.46	52.45 ± 25.36	-2.285^a^
SF-36 Role-emotional	27.03 ± 35.87	52.17 ± 41.37	-2.918^b^
SF-36 Mental Health	43.89 ± 18.07	60.00 ± 17.30	-4.133^c^
SF-36 Health Change	70.27 ± 27.54	64.13 ± 27.71	1.006

**Categorical**	***n* (%)**	***n* (%)**	**χ^2^ statistic**

Marital status			2.112
single	1 (2.7)	5 (10.9)	
married	29 (78.4)	34 (73.9)	
divorced	6 (16.2)	6 (13.0)	
widow	1 (2.7)	1 (2.2)	
Working status			2.319
Student	1 (2.8)	0	
Employed	19 (52.8)	26 (57.8)	
Unemployed	2 (5.6)	2 (4.4)	
Retired	5 (13.9)	9 (20.0)	
Housewife	9 (25.0)	8 (17.8)	
Missing data	1	1	


*FM, fibromyalgia; TCI, Temperament and Character Inventory; FIQ, Fibromyalgia Impact Questionnaire; HADS, Hospital Anxiety and Depression Scale; and SF-36, Short Form Health Survey 36. ^a^*p*-value < 0.05. ^b^*p*-value < 0.005. ^c^*p*-value < 0.001.*

### Evaluation of the Quality of Cluster Analysis

Discriminant function analysis assumptions of independence of observations, multivariate normality and absence of multicollinearity were met. Box’s test of equality of covariance ascertained homoscedasticity (Box’s *M* = 37.457 and *p*-value = 0.203). The model was statistically significant (Wilk’s = 0.290, χ^2^(7) = 95.915, and *p*-value < 0.001) and provided a canonical correlation of 0.843 that accounted for 70% of the total variance (0.843^2^ = 0.711). **Table 3** reports the predictors’ loadings and shows the consistency between the discriminant function coefficients and the correlation coefficients. HA and SD were the best predictors of cluster membership. The model also showed satisfactory accuracy in prediction, correctly classifying 96.4% of the cases (prediction rates of 89.2% for Cluster1 and 97.8% for Cluster2).

**Table 3:** Discriminant analysis summary: function coefficients and correlation coefficients.

Predictor	SCDFC	Predictor	SM
TCI HA	-0.609	TCI SD	0.667
TCI NS	0.279	TCI HA	-0.637
TCI RD	0.312	TCI C	0.319
TCI P	-0.083	TCI RD	0.173
TCI SD	0.775	TCI NS	0.125
TCI C	0.001	TCI P	-0.082
TCI ST	0.007	TCI ST	-0.013


*TCI, Temperament and Character Inventory; SCDFC, standardized canonical discriminant function coefficients; and SM, structure matrix.*

## Discussion

Overall, the findings from the present study showed a particular personality for FM patients and suggested two different clusters in terms of both personality traits and clinical characteristics, as proposed by Gonzalez et al. (2015), confirming the importance of the personality assessment of FM patients (Malin and Littlejohn, 2012).

Regarding the personality, in comparison with the general population and the CG enrolled in the study, FM patients were characterized by the tendency to be fearful, apprehensive, discouraged, insecure, negativistic (due to their higher levels on HA), slow tempered, slow to engage, unenthusiastic (due to their lower levels on NS), less mature, less responsible, blaming, and driven to react to current circumstances and immediate needs (due to their lower levels on SD). Our observations about the peculiar personality traits of FM patients corroborate findings from previous studies (Mazza et al., 2009; Santos Dde et al., 2011; Gencay-Can and Can, 2012; Garcia-Fontanals et al., 2014) and similar results in studies conducted on patients affected by other psychosomatic diseases using TCI (Boz et al., 2004; Mongini et al., 2005; Kim et al., 2006; Malmgren-Olsson and Bergdahl, 2006; Conrad et al., 2007; Kiliç et al., 2008). These studies often reported high HA and low SD levels in psychosomatic patients. From a biopsychosocial perspective, HA, as a temperament trait, could be considered amongst the biological factors involved in the pathogenesis of psychosomatic syndromes. Such an holistic approach, as the psychobiological model of personality, could be useful to explore the determinants of health and disease from an integrated point of view. Concerning FM, these considerations raise the question of whether the personality traits are the cause or effect of the syndrome. A high HA temperament trait may be considered a significant predictor of FM; at the same time, a painful chronic condition may lead to avoidant behaviors. Current knowledge on this issue does not allow us to draw a conclusion; further studies are needed to clarify the role of HA in FM. This issue is particularly important considering that HA influences the perception of pain. In fact, harm avoidant people show an increased response to pain (Pud et al., 2004). Although previous studies have found that neither HA nor SD personality traits correlate with the pain characteristic of FM (Gencay-Can and Can, 2012; Garcia-Fontanals et al., 2014), other evidence has demonstrated that the higher the score on HA, the higher the perceived pain intensity experienced by the patient (Garcia-Fontanals et al., 2014). This latter evidence suggests that HA could influence the rating of intensity of a pain stimulus by lowering the threshold at which pain is perceived as threatening. SD could play the opposite role: the higher the score on SD, the lower the perceived pain intensity experienced by the patient (Garcia-Fontanals et al., 2014). Therefore, a personality characterized by high HA and low SD could significantly discourage the patient from dealing with stressful situations, such as a chronic pain condition (Garcia-Fontanals et al., 2014). This hypothesis seems to be sustained by recent observations about the impact of character dimensions, such as SD, on the self-perceived wellness and happiness (Cloninger and Zohar, 2011). As foundation of Self-regulation (i.e., sense of self-efficacy and self-determination), SD may influence hopes and beliefs about perceived health and sickness status (Cloninger et al., 1994; Cloninger and Zohar, 2011).

Regarding the findings that emerged from the cluster analysis, two different subgroups of FM patients were identified. Cluster1 was marked by the presence of the aforementioned personality characteristics more than Cluster2 and presented a more severe symptomatology. From these preliminary observations, we can argue that the FM population seems to be composed of a subgroup of patients (Cluster1) that is more vulnerable to health-related distress, anxiety, mood and personality disorders (mainly due to their higher levels of HA, around the 98th percentile rank, and their lower levels of SD, around the 15th percentile rank) and an other subgroup of patients (Cluster2) with better health-related quality of life and less severe fibromyalgic and psychiatric symptomatology, probably due to the protective role of a mature character that allows them to better address stressful life events (Svrakic et al., 1993). Our observations seem to confirm previous evidence from a different theory of personality (i.e., the Five-Factor Model), in which the clusters identified differ from one another in terms of neuroticism and extraversion (Torres et al., 2013), two dimensions that broadly overlap the conceptualization of HA and SD (De Fruyt et al., 2000). As also suggested by Torres et al. (2013) regarding their own findings, the clusters we identified in the present study may represent more than the consequence of splitting patients according to the severity of their symptoms. The clusters were identified starting from personality traits and may reflect differences in sensitivity to stimuli (an aspect of temperament) and in psychological functions, such as the emotional processing of somatic perceptions such as pain. Evidence on the associations between alexithymia and increased affective pain and hypochondriacal illness behavior among FM patients seems to support this hypothesis (Huber et al., 2009; Di Tella and Castelli, 2013).

All the findings discussed gain importance if level of FM patients’ emotional distress is considered. Results from this study showed subclinical levels of emotional distress for Cluster2 but borderline clinical anxiety and depression for Cluster1. This could mean that a relevant quote of FM patients may be treated for psychiatric morbidities and, considering the aforementioned interactions between personality dispositions and emotional reactions, a complete assessment of aspects of temperament and character seems to be essential in FM treatment and care.

The present study contains several limitations, the most significant of which is represented by the small sample size. Although discriminant function analysis provides an acceptable index of the goodness of fit of cluster analysis in conditions of small sample size, the sampling adequacy of the present study is not optimal, and the statistical power of the analysis is affected. Therefore, the findings of the study must be considered preliminary and not generalizable. Future research could replicate this study with a larger sample in different contexts. A second limitation is represented by the self-assessment procedure of data gathering used in the study. If we assume the possibility of alexithymia in FM patients, we have to take into account the gap between the observed responses and the actual condition of the patients due to their difficulty in identifying feelings and somatic perceptions. Further studies could address this issue by using different research methodologies or other procedures of data collection.

Despite the aforementioned limitations, the findings of the present study seem to confirm the presence of peculiar personality dimensions in the FM population, in which HA and SD play an important role. Higher HA and lower SD allow us to identify a subgroup of FM patients with more severe symptomatology. These results suggest that personality assessment could be a useful step in the diagnostic process of FM patients and could allow clinicians to optimize therapeutic intervention by placing patients into different therapeutic programs depending on their personality characteristics.

## Author Contributions

PL and FZ: Project design; MM: data analysis; PL, MM, and FZ: paper writing; FZ, FC, and EF: participants recruitment and testing; LC and RT: paper revision; and PL, RT: project supervision.

## Conflict of Interest Statement

The authors declare that the research was conducted in the absence of any commercial or financial relationships that could be construed as a potential conflict of interest.
